# On the Creation of Representative Samples of Random Quasi-Orders

**DOI:** 10.3389/fpsyg.2015.01791

**Published:** 2015-11-27

**Authors:** Martin Schrepp, Ali Ünlü

**Affiliations:** ^1^SAP AGWalldorf, Germany; ^2^Technical University of Munich (TUM), Centre for International Student Assessment (ZIB)Munich, Germany

**Keywords:** learning space theory, item tree analysis, quasi-order, representative sampling, inductive uniform extension

## Abstract

Dependencies between educational test items can be represented as quasi-orders on the item set of a knowledge domain and used for an efficient adaptive assessment of knowledge. One approach to uncovering such dependencies is by exploratory algorithms of *item tree analysis* (ITA). There are several methods of ITA available. The basic tool to compare such algorithms concerning their quality are large-scale simulation studies that are crucially set up on a large collection of quasi-orders. A serious problem is that all known ITA algorithms are sensitive to the structure of the underlying quasi-order. Thus, it is crucial to base any simulation study that tries to compare the algorithms upon samples of quasi-orders that are representative, meaning each quasi-order is included in a sample with the same probability. Up to now, no method to create representative quasi-orders on larger item sets is known. Non-optimal algorithms for quasi-order generation were used in previous studies, which caused misinterpretations and erroneous conclusions. In this paper, we present a method for creating representative random samples of quasi-orders. The basic idea is to consider random extensions of quasi-orders from lower to higher dimension and to discard extensions that do not satisfy the transitivity property.

## Introduction

Orders play an important role in various formal theories of behavioral, social, economic, or computer sciences. Examples are in decision making and preference modeling (e.g., Fishburn, 1972; Peterson, 2011) or economics (e.g., Varian, 2002). Other areas where order relations play an important role are in computer science, for example in database systems research (e.g., Rob and Coronel, 2009). There are also several applications that try to set up a structure that represents common beliefs of respondents in the form of an order relation in sociological questionnaires (e.g., Wiley and Martin, 1999; Martin and Wiley, 2000; Schrepp, 2005).

First and foremost, we are interested in *quasi-orders* or *preorders* (i.e., reflexive and transitive binary relations) as a cornerstone concept in the educational or psychological theory of *knowledge* or *learning spaces* (Doignon and Falmagne, 1985, 1999; Falmagne and Doignon, 2011; Falmagne et al., 2013). Knowledge or learning space theory interprets discrete order structures such as the quasi-orders in the human-centered knowledge or competence modeling and assessment context. The basic idea underlying the theory is to formalize the adaptive approach of a teacher, when the teacher's experience and knowledge about prerequisite relations between the pieces of knowledge (e.g., algebra questions) are utilized to avoid asking a student questions neither too easy nor too difficult, so to operate in this way at the borderline of what the student masters and what he does not know.

Prerequisite relations can be modeled using quasi-orders. In knowledge or learning space theory, a quasi-order ≤ on a test or item set *Q* is also called a *surmise relation* because it has the following interpretation. Typically, the items represent problems or questions, for example from the domains of mathematics or science, which the subjects can solve or fail to solve. A prerequisite relation (implication or dependency) *i* ≤ *j* is interpreted as stating “*Each subject who is able to solve item j is also able to solve item i.”* As an example, a plausible dependency to postulate between the two algebra items “*a. Perform the multiplication 4x*^*4*^*y*^*4*^· *2x* · *5y*^*2*^ *and simplify”* and “*b. Find the greatest common factor of the expressions 14t*^*6*^*y and 4tu*^*5*^*y*^*8*^ *and simplify”* is to assume that the mastery of problem *a* is a prerequisite for the mastery of problem *b*. Thus, the items *a* and *b* are in relation with respect to a quasi-order ≤, denoted by *a* ≤ *b*.

Quasi-orders representing such item dependencies can be derived by querying experts or from postulated theoretical assumptions (e.g., Albert and Lukas, 1999), or by exploratory data analysis methods such as *item tree analysis* (ITA; Van Leeuwe, 1974; Schrepp, 1999, 2003; Sargin and Ünlü, 2009). In fact, the goal of any ITA method is to reconstruct, by data analysis of a collection of observed noisy response patterns, the underlying or true dependencies among the items, and therefore, the ITA methods can also be utilized for validating expert judgments or theories that imply subjective or theoretical item hierarchies, respectively. However, developing, evaluating, and comparing ITA-type algorithms used to extract true relational dependencies or surmise relations from sets of observed response patterns are a challenging task and an important branch of research.

A specific problem in this respect is addressed in the present paper. We deal in this work with the question of how random quasi-orders can be generated, for example as a basis for simulation studies to investigate the performance or properties of such data analytical methods, or indirectly to validate, in the sense we described above, possible theories or expert judgments. As discussed by Ünlü and Schrepp (2015), ITA simulation studies do create response data from a given quasi-order, by simulating random response errors, and then do check if the original quasi-order can be reconstructed, by analyzing the simulated data based on a data analysis method. Since all known ITA algorithms are sensitive to the structure of the quasi-order, it is important to use a representative set of quasi-orders as the basis for the simulations. Using non-representative quasi-order samples yielded biased or erroneous simulation results regarding the recovery quality of the algorithms (Ünlü and Schrepp, in press).

For the purpose of reconstructing mastery hierarchies among items several ITA algorithms have been published in the last years (Van Leeuwe, 1974; Schrepp, 1999, 2003, 2006; Sargin and Ünlü, 2009, 2010; Ünlü and Sargin, 2010). Large-scale simulation studies are conducted to compare such algorithms concerning their ability to detect a surmise relation. More precisely, these studies start with a sample of surmise relations on the item set *Q*, and then create, for each of the posited surmise relations, a data set *D* (for details see e.g., Ünlü and Schrepp, 2015):

- The surmise relation ≤ determines the set of response patterns compatible with all of the dependencies in ≤.
- Then random response errors, for example lucky guesses or careless errors, are simulated.
- This data set *D* is analyzed with the algorithm, and it is checked how close the resulting surmise relation is to the underlying surmise relation used to generate the data set.

We see that at the basis of this type of simulation study is a large set of quasi-orders assumed to underlie an item set (Schrepp, 2007). A serious problem is that all known ITA algorithms are sensitive to the structure of the underlying surmise relation. For example, the original procedure by Van Leeuwe (1974) works very well for linear orders, but is unsatisfactory for quasi-orders that contain many non-comparable item pairs *i* ≰ *j* or *j* ≰ *i* (see e.g., Schrepp, 1999). Therefore, it is essential to base any simulation study that aims at comparing different ITA-type algorithms on samples of quasi-orders that are representative, in the sense that each quasi-order on the item set is included in a sample with the same probability.

Let *X* be a non-empty, randomly generated subset of quasi-orders on a set *Q* of *n* items (below we will outline different approaches to creating quasi-order samples). We call *X* a *representative* subset or sample if *P*(≤_1_ ∈*X*) = *P*(≤_2_ ∈*X*) for any two quasi-orders ≤_1_ and ≤_2_ on *Q*, where *P* denotes probability. Since this definition is for arbitrary quasi-orders, as a corollary, we obtain that any such representative set *X* of quasi-orders must also be “*proportionally representative”* for the population distributions of quasi-order size, quasi-order width, and quasi-order height (defined below), in the sense that the corresponding sample distributions computed in *X* provide unbiased estimates of their population analogs. The latter distributional properties are the evaluation criteria used for assessing the extent of representativeness in this paper.

In Ünlü and Schrepp (2015, in press), it was shown that conclusions concerning the performance of the variants of ITA in earlier publications were biased due to the use of non-representative samples of underlying quasi-orders. By repeating simulations with a representative sample drawn from the set of all quasi-orders on six items, the biased results could be corrected, and a clear picture of the performance of the ITA algorithms was reached. The maximum number of items used in the latter study was six, because higher item numbers were prohibitively too large for realizing representative samples. With the present paper, we introduce an algorithm that allows constructing large samples of representative quasi-orders efficiently, and on larger item sets.

In the next section, we discuss existing approaches to addressing this problem and the critical issues related with these. In a next step, the description of the proposed algorithm is given, and then simulation results are presented demonstrating the usefulness of the sampling procedure. Finally, we conclude with a summary and further related remarks.

## Existing approaches to creating random quasi-orders

Formally, the problem we address in this paper can be described as follows. Presuppose a set *Q* of *n* test items. The goal is to create randomly a sample, PQO(*n*), of quasi-orders that is representative, in the sense that each quasi-order on *Q* is included in the sample with the same probability.

We use the relational matrix notation, i.e., a binary relation *R* on *Q* (not necessarily reflexive or transitive) is denoted by a matrix (*r*_*ij*_) with *r*_*ij*_∈ {0, 1} and *r*_*ij*_ = 1 ⇔ *iRj*.

In this notation, reflexivity and transitivity of any binary relation *R* mean that its corresponding relational matrix satisfies the following properties:

- *r*_*ii*_ = 1 for all *i* = 1, …, *n* (reflexive *R*),
- (*r*_*ij*_ = 1 ∧ *r*_*jk*_ = 1 → *r*_*ik*_ = 1) for all *i,j,k* ∈ {1*, …, n*} (transitive *R*).

There are two direct methods to create a representative sample of quasi-orders. First, simply construct and store all quasi-orders on *Q* and then select the sample by a random process that draws elements from the set of all quasi-orders with the same probability. This does not work even for small values of *n*, since the number of quasi-orders on a set of *n* items grows very fast with *n*. For example, on a set of three items there are 29 quasi-orders, on six items this number already grows to 209, 527 (e.g., Pfeiffer, 2004).

Another direct approach is to create a huge number of random reflexive relations on *n* items and to keep the transitive ones. This process can be described as follows:

- Create a relational matrix (*r*_*ij*_), where *r*_*ii*_ = 1 for all *i* = 1, …, *n*, and the other values *r*_*ij*_ are chosen with equal probability from {0, 1}.
- Check if the generated relation is transitive. If this is the case, add it to the sample. Otherwise discard it, go back to the previous step and create again a random reflexive relation on *n* items.
- This process continues until the required number of quasi-orders contained in the sample is attained.

This method, directly operating on all items simultaneously, also fails even for moderate sizes of *n*, since the chance that a randomly created reflexive relation is transitive becomes extremely small. For *n* items, we have 2^*n*^2^^−*n* reflexive relations, since each reflexive relation can be represented by an *n*×*n* matrix containing 1 in the cells of the diagonal and one of the values 0 or 1 in all of the other *n*^2^−*n* cells. If we compare these numbers for *n* = 3, …, 6 with the numbers of (labeled) quasi-orders, we can see in Table 1 that even for such small *n* the chance to pick a transitive relation using this random process is tiny.

**Table 1:** **Probability to detect a quasi-order by random selection**.

***n***	**Number of reflexive relations**	**Number of quasi-orders**	**Selection probability**
3	2^6^ = 64	29	0.453
4	2^12^ = 4096	355	0.087
5	2^20^ = 1, 048, 576	6942	0.007
6	2^30^ = 1, 073, 741, 824	209, 527	0.0002

Thus, finding for example 10,000 quasi-orders on *n* = 6 items requires to select ~50,000,000 random reflexive relations, i.e., 1,500,000,000 random 0–1-numbers. If we would be able to generate and check 1000 reflexive relations per second, it would require ~50,000 s until the process stops. It is obvious that this will not work at all for larger item numbers, for example for *n* = 10. In contrast, the processing times of the newly proposed procedure are feasible and the results can be found in the section Creating Quasi-orders on Bigger Item Sets.

Previous studies tried to avoid this problem by implementing *ad-hoc* procedures to draw random quasi-orders. Although these methods are very flexible and virtually work for any value of *n*, they lack representativeness in their generation processes. The simplest method is to create a random relation on *n* items and to compute the transitive closure of this relation. It was found in earlier publications (e.g., Sargin and Ünlü, 2009) that this simple strategy produced samples of quasi-orders, which were far from being representative concerning quasi-order sizes.

Some more advanced strategies tried to compensate this issue. For example, in Sargin and Ünlü (2009) the following procedure was used. Generate a relational matrix (*r*_*ij*_) such that:

1. *r*_*ii*_ = 1 for all *i* = 1, …, *n*.
2. For all *r*_*ij*_ with *i*, *j* = 1, …, *n* and *i* ≠ *j*, the value *r*_*ij*_ is chosen randomly with probabilities *P*(*r*_*ij*_ = 1) = *x* and *P*(*r*_*ij*_ = 0) = 1−*x*. The probability *x* itself is a realization of a normal distribution with parameters μ = 0.16 and σ = 0.06. Values “< 0” or “> 0.3” are set to 0 or 0.3, respectively^1^.
3. The transitive closure of the binary relation corresponding to (*r*_*ij*_) is the resulting random quasi-order.

**Figure 1:**
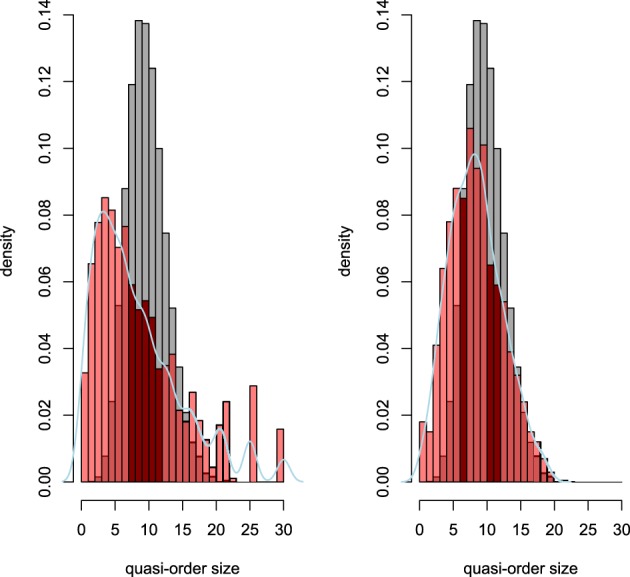
**For six items, in red, histogram densities of quasi-order size (i.e., number of item pairs in relation) for 10,000 (left panel) and 100,000 (right panel) quasi-orders randomly created according to the two variants of the normal procedure**. In addition, kernel density estimates of the samples are plotted, in light blue, to assist visualization. In gray, the true distribution is shown, with overlapping areas of the true and sampled printed in dark red.

This random process—existent in two variants, absolute and averaged; for details, see Sargin and Ünlü (2009)—is already an improvement of an older procedure (Schrepp, 1999) that drew *x* based on a uniform distribution on the interval 0–0.4 and that resulted in non-representative samples consisting of overly represented large quasi-orders. But this improved normal procedure still produced non-representative samples, as can be seen from Figure 1 (Ünlü and Schrepp, 2015).

## Description of the algorithm

The algorithm is inductive, i.e., a procedure for creating a representative set of quasi-orders on *n* + 1 items, on the basis of a prior constructed set of quasi-orders on *n* items.

In principle, we can always start the process, the anchoring, with the set of all quasi-orders for a sufficiently small value of *n*.

For the step from *n* to *n* + 1, we consider a set PQO(*n*) of *m* quasi-orders on *n* items and assume that PQO(*n*) is representative, in the sense that each quasi-order on the *n* items is contained in this set with the same probability. Thus, PQO(*n*) can be seen as a random sample drawn from the set of all quasi-orders on *n* items. In the induction step, we create for each quasi-order in PQO(*n*), subsequently represented by its relational matrix (*q*_*ij*_), a number of *z* random extensions to *n* + 1—i.e., each *random extension* of (*q*_*ij*_), denoted as the matrix ($q_{ij}^{\prime }$), is defined by:

- $q_{ij}^{\prime }=q_{ij}$ for *i*, *j* = 1, …, *n*,
- $q_{n+1n+1}^{\prime }=1$ (reflexive extension),
- for indices *i* ≠ *j* with *i* = *n* + 1 or *j* = *n* + 1, $q_{ij}^{\prime }$ is a random variable with $P\left(q_{ij}^{\prime }=1\right)=P\left(q_{ij}^{\prime }=0\right)=0.5$.

The *z* random extensions created are checked for transitivity, and the transitive ones are added to PQO(*n* + 1). Duplicates are removed, i.e., each quasi-order occurs only once in PQO(*n* + 1). Regarding the choice of *z*, note that the chance a random extension of a quasi-order on *n* items again yields a quasi-order on *n* + 1 items decreases with increasing item number *n*. Thus, higher values for *z* must be used when *n* is getting larger, and in particular, this number must be adjusted depending on the number of quasi-orders that should be created during simulation. A general strategy easily possible is to specify *z* in a way such that slightly more quasi-orders than desired are generated. In a final step of the procedure, PQO(*n* + 1) is reduced to a required or reasonable size, for example by keeping only *m* randomly selected elements from PQO(*n* + 1) based on simple random sampling. This step is necessary to have the number of elements of the PQO(*n*)'s reasonably limited, if this process is repeated several times; for instance, when creating a sample consisting of 1000 quasi-orders on 10 items starting with the set of all four quasi-orders on two items.

This inductive algorithm produces representative samples of quasi-orders.

**Proposition**. Let ≤_1_ and ≤_2_ be any two quasi-orders on *n* + 1 items. Denote by *P*_1_ and *P*_2_ the probabilities that ≤_1_ and ≤_2_ are contained in the set PQO(*n* + 1) that is generated according to the proposed inductive procedure, respectively. Then, *P*_1_ = *P*_2_.

**Proof**. The proof is by induction. As its anchoring, the algorithm always starts with a representative set of quasi-orders for a sufficiently small item number *l*. We consider the inductive step from *n*≥*l* to *n* + 1. Let ≤_*i, n*_ be the *traces* of ≤_*i*_ (*i* = 1, 2) on the *n* items of the predecessor construction step, that is, the quasi-orders restricted to those *n* items. Since PQO(*n*) constructed in the predecessor step *n* is assumed to be representative in the sense we defined (the induction hypothesis), the probabilities for the two traces to belong to PQO(*n*), in respective order, *P*_1, *n*_ and *P*_2, *n*_, are the same. Moreover, all random extensions (to the *n* + 1 items) of any given trace quasi-order (on the n items) are equally likely, with the same probability of 2^−2*n*^ and independent of the trace quasi-order considered. Thus, $P_{1}=2^{-2n}P_{1,n}=2^{-2n}$ *P*_2, *n*_ = *P*_2_.

## A first simulation study

To assess the quality of the algorithm used to create random quasi-orders we performed a first simulation study. In the study, we generated 100 random samples each of (sample sizes) 100, 1000, and 5000 quasi-orders on a set of six items, which were compared with the set of all possible quasi-orders concerning their distributions of important properties.

We use the following properties as the criteria for representativeness of the samples.

1. **Size**: The size of a quasi-order ≤ is defined as the number of item pairs (*i, j*) with *i* ≤ *j*.
2. **Width**: The size of a longest anti-chain in ≤ : An *anti-chain* is a subset of items {*i*_1_, …, *i*_*k*_} with ¬(*i*_*x*_ ≤ *i*_*y*_) for all *x, y* ∈ {1, …, *k*} (¬, the negation).
3. **Height**: The size of a longest chain in ≤ : A *chain* is a subset of items {*i*_1_, …, *i*_*k*_} with *i*_1_ ≤ *i*_2_∧ …∧ *i*_*k*−1_ ≤ *i*_*k*_.

The sampling process started with the set of all four quasi-orders on a set of two items. The inductive procedure described in the last section was then employed to create random quasi-orders on *n* = 6 items. The computations reported in this paper were done with a C program that implements the described algorithm on a computer with an Intel Core i5 2.50 GHz processor.

If we compare the mean values of the average sizes, widths, and heights taken over the 100 generated samples, we see that the results approximate the true values calculated for the set of all quasi-orders on six items very well (see Table 2).

**Table 2:** **Means and standard deviations (in parentheses) of the average sizes, widths, and heights over all 100 simulated samples of 100, 1000, and 5000 quasi-orders (PQO's), compared to the true average values for the set of all quasi-orders on *n* = 6 items**.

	**100 PQO's**	**1000 PQO's**	**5000 PQO's**	**True value**
Mean size	15.15 (0.35)	15.13 (0.20)	15.20 (0.16)	15.22
Mean width	2.64 (0.07)	2.64 (0.04)	2.63 (0.03)	2.62
Mean height	3.60 (0.11)	3.60 (0.06)	3.62 (0.04)	3.62

**Figure 2:**
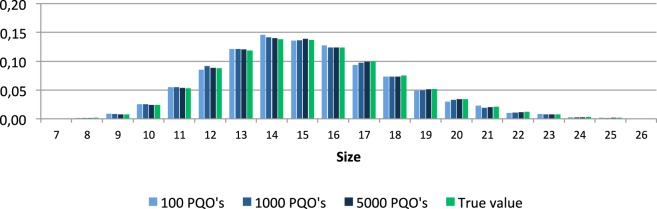
**Distributions of the relative sizes in the set of all quasi-orders and corresponding mean values of the relative sizes in the 100 simulated quasi-order samples (PQO's)**.

Figure 2 shows the distributions of the relative sizes for the set of all quasi-orders and of the mean values of the relative sizes computed over all 100 generated samples.

**Figure 3:**
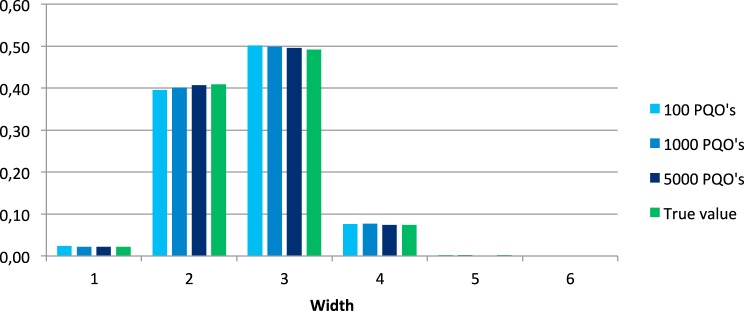
**Distributions of the relative widths in the set of all quasi-orders and corresponding mean values obtained from the 100 simulated samples of 100, 1000, and 5000 quasi-orders (PQO's)**.

**Figure 4:**
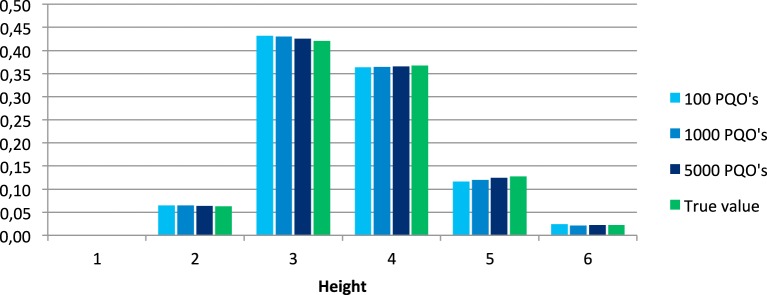
**Distributions of the relative heights in the set of all quasi-orders and corresponding mean values obtained from the 100 simulated samples of 100, 1000, and 5000 quasi-orders (PQO's)**.

Figures 3, 4 show the distributions of the relative widths and heights for the set of all quasi-orders and of the mean values of the relative widths and heights over all 100 generated samples.

## Creating quasi-orders on bigger item sets

In a second simulation study, we investigated how much resources were needed to create representative samples of quasi-orders on bigger item sets.

The study started with a sample of 10,000 quasi-orders that was drawn randomly from the set of all 209, 527 quasi-orders on six items (see Ünlü and Schrepp, 2015).

From this set, samples of quasi-orders on *n* = 7, …, 20 items were constructed inductively.

**Figure 5:**
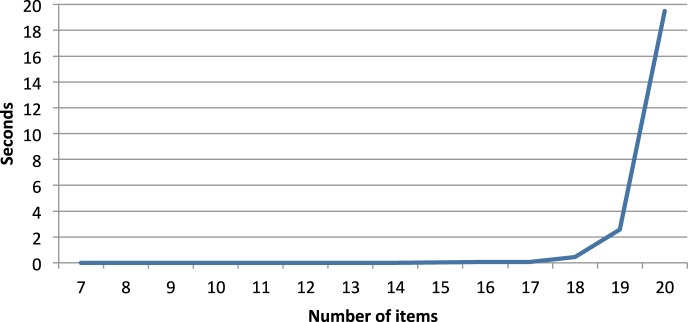
**Required computing time (in s) per produced quasi-order for different numbers of items**.

Figure 5 shows how the computing time in s (per quasi-order) required to produce the quasi-orders evolves. The exact values can be seen in Table 3.

**Table 3:** **Required computing time in s and used number of random 0–1-values per produced quasi-order**.

***n***	**Computing time (in s)**	**Number of random 0–1-values**
7	1.4809E-05	1039
8	2.65824E-05	3243
9	5.70143E-05	10,470
10	0.00013198	32,479
11	0.000334222	99,200
12	0.000904816	300,011
13	0.003017408	960,431
14	0.011056273	3,192,934
15	0.039129196	10,750,095
16	0.059797056	23,245,005
17	0.077668647	32,748,257
18	0.458117766	222,208,059
19	2.573514793	1,163,076,923
20	19.49241959	9,388,888,889

As can be seen, we still have acceptable computing times up to 17 items, but then the required computing time grows fast. The exponential increase of computing time obviously limits the possible scope of the method.

**Figure 6:**
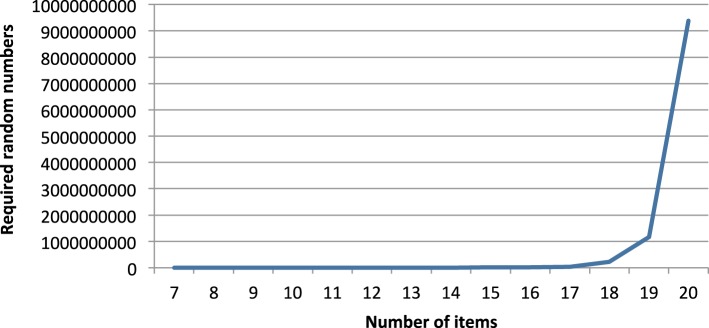
**Required random 0–1-numbers per produced quasi-order for different numbers of items**.

Figure 6 shows the numbers of random 0–1-values that are required on average to produce one quasi-order, reported for the different item counts. The exact values can be found in Table 3.

Thus, highly efficient processes to produce random 0–1-numbers are required to apply the algorithm. Obviously, the current limit on applications of the algorithm running on standard machines will be around 15–20 items, as long as we will not accept runtimes of several days. But since the algorithm can easily run in parallel architectures, i.e., on several computers in parallel or on multi-processor machines, this limit can be increased.

An interesting point is to compare the performance of the new algorithm with the performance of the basic approach that directly creates random reflexive relations and checks if they are transitive (see Table 1). For *n* = 6, the chance that a randomly generated reflexive relation is a quasi-order is 0.0002, and to obtain such a random reflexive relation we need to have 30 random 0–1-values. Now compare this to the new procedure, for the step from 10 to 11 items. Creating a random reflexive extension on 11 items, of a quasi-order on 10 items, requires 20 random 0–1-values. The probability for such a random extension to satisfy transitivity is 0.0002, i.e., as high as the probability to generate a random quasi-order on only six items using the basic or direct method. Thus, working with successive extensions of lower-dimensional quasi-orders to higher dimension massively increases the probability for a random reflexive extension to be transitive too, and reduces the number of necessary random 0–1-values, compared to the direct or purely random generation of binary relations.

## Conclusion

Algorithms that create quasi-orders by exploratory data analysis are a relevant research topic in knowledge or learning space theory. Typically, the quality of such algorithms cannot be evaluated on the basis of purely theoretical arguments. Large-scale simulation studies are required to examine whether such algorithms can handle different quasi-order structures underlying the data and various ranges of simulated response error probabilities.

Representative samples of quasi-orders are a prerequisite for such studies. However, up to now it has not been possible to create truly representative samples for larger item numbers. Thus, previous simulation studies had to live with approximations, which in some cases had a negative impact on the simulation results and caused biased or incorrect conclusions (Ünlü and Schrepp, 2015, in press). The only possible workaround was to draw samples of quasi-orders from the prior constructed set of all quasi-orders, which merely worked for small item sets.

We have described an inductive algorithm that allows creating representative samples of quasi-orders even for higher item numbers in still acceptable runtime. The algorithm successively considers random reflexive extensions of lower dimensions to higher. In one simulation study with six items, we have seen that the results regarding quasi-order size, width, and height do approximate the true values very well. In a further simulation study, we have also investigated generating representative samples of quasi-orders on bigger item sets. Although the required computing time increases relatively fast, we still have obtained acceptable processing times for up to 17 items.

The problem considered in this paper is a very specific one, namely, to provide a sound basis for the reliable comparison of the ITA algorithms in simulation studies. For this purpose, the studied range of 10–20 items is adequate. However, it could be interesting to develop, in future research, extensions of the proposed technique that allow sampling representative quasi-orders on more than 20 items. This may be useful, for instance, in applications to international educational large-scale assessments such as PISA or TIMSS (e.g., Ünlü et al., 2014), although in that context smaller subtests or item collections of appropriate sizes could also be studied.

Representative samples of quasi-orders can be employed in subsequent research to investigate the properties of and thereby improve on data analysis methods used to mine for relational dependencies in psychological or educational response data, since properties investigated in representative samples can be generalized to the population of all quasi-orders.

In principle, the methods that we have discussed could generally be applied in other fields or situations (cf. also Section Introduction). For example, linear orders are a special case of quasi-orders and, as pointed out by Augustin Kelava, may be useful in the analysis of item position or item order effects in large-scale assessments. Knowledge or learning space theory combinatorial structures or computational ITA analyses could be utilized to study these effects. Future research into this issue is needed.

### Conflict of interest statement

The authors declare that the research was conducted in the absence of any commercial or financial relationships that could be construed as a potential conflict of interest.

## Footnotes

^1^Strictly speaking, there is no driving force for the use of the normal distribution. For example, other symmetric distributions with direct location and scale measures could also be used. It is worth mentioning, however, that the distributions of quasi-order size seem to roughly follow a bell-shaped curve, deeming the normal choice a plausible one. Moreover, it is obvious that different values of *x* are needed to simulate sufficiently varying quasi-order sizes. More importantly, note that this procedure is *ad-hoc*, and this is the reason why we propose a principled new approach.
